# Branched Hyaluronic Acid for Reduced Viscosity and Enhanced Moisturization

**DOI:** 10.3390/ma18214882

**Published:** 2025-10-24

**Authors:** Hyun Ji Lee, In Young Lee, Yongseok Choi, Yun-chan Lee, Kuen Yong Lee

**Affiliations:** 1 Department of Bioengineering, Hanyang University, Seoul 04763, Republic of Korea; guswl331-@daum.net (H.J.L.); 96dlsdud@naver.com (I.Y.L.); junnyjack12@naver.com (Y.C.); harryyunchan@naver.com (Y.-c.L.); 2 Institute for Bioengineering and Biopharmaceutical Research, Hanyang University, Seoul 04763, Republic of Korea

**Keywords:** hyaluronic acid, branched structure, viscosity, moisturizing effect

## Abstract

Despite its remarkable moisturizing properties, the inherently high viscosity of high-molecular-weight hyaluronic acid (HA) restricts its practical application in skincare products, cosmetic formulations, and skin-contact medical devices. To overcome this limitation, we propose the incorporation of branched structures into HA to create a branched HA hybrid (bHH) by chemically coupling low-molecular-weight HA (200 kDa) with high-molecular-weight HA (700–2500 kDa). The introduction of branched structures into the HA backbone alters the viscosity of high-molecular-weight HA while preserving its moisturizing potential. Branching reduces the solution viscosity of linear HA, particularly at higher polymer concentrations. In this study, the moisturizing efficacies of branched and linear HAs were extensively evaluated. Branched HA demonstrated equivalent or superior moisturizing effectiveness compared with linear HA and even significantly enhanced the water-binding capacity over simple mixtures of linear HAs. These findings suggest that introducing branched structures can effectively reduce the solution viscosity of linear HA without compromising its moisturizing properties, thereby improving the usability and hydration performance of skincare products and skin-contact devices.

## 1. Introduction

Moisturizing agents are essential components in dermatological and cosmetic care, playing a crucial role in maintaining optimal skin hydration, preserving barrier function, and supporting overall skin integrity and health [1]. These agents consist of various ingredients, including occlusives, humectants, preservatives, and other active substances, designed to hydrate and nourish the skin. This improves its ability to retain moisture, and they are generally divided into small molecules and macromolecules, each with distinct mechanisms, advantages, and limitations [1,2]. Humectants, such as glycerin, propylene glycol, polyhydric alcohols, and organic salts, draw water to the skin, keeping it smooth and soft [2,3]. Glycerin is valued for its low cost and effectiveness [4], while urea and lactic acid hydrate and gently exfoliate, supporting natural skin renewal [5]. However, small molecule typically offers relatively transient hydration because of its low molecular-weight (MW) and rapid evaporation or absorption, which necessitates frequent reapplication. Effective humectants must exhibit several key properties, such as skin compatibility, high water-retention efficiency, appropriate viscosity, and desirable sensory attributes [6,7].

Hyaluronic acid (HA) is a widely used humectant because of its excellent water-binding capability of up to 1000 times its own weight in water [8,9]. Structurally, HA is composed of repeating disaccharide units of N-acetyl-D-glucosamine and D-glucuronic acid [10,11], contributing significantly to various physiological processes, including skin hydration, wound healing, and maintenance of extracellular matrix integrity [12,13]. Furthermore, HA has been widely used to prepare synthetic extracellular matrices for various tissue engineering applications, including wound repair, bone regeneration, ischemia treatment, and corneal repair [14], owing to its excellent biocompatibility and nontoxic degradation products [15,16]. The topical application of HA is well-known to enhance skin hydration and elasticity while minimizing the appearance of fine lines and wrinkles, contributing to a more youthful appearance [17,18,19].

The hydration capability of HA generally increases with concentration, and it is typically used at a concentration range from 0.2% to 1.0% (*w*/*w*) in cosmetic formulations [8,20]. HA is classified by MW, with <200 kDa as low-molecular-weight HA (Low-MW HA) and >1000 kDa as high-molecular-weight HA (High-MW HA) [21]. Low-MW HA rapidly penetrates the skin, producing a transient improvement in hydration; however, this effect is short-lived, and higher concentrations lead to increased viscosity [22,23]. High-MW HA forms a continuous hydrophilic film on the skin surface, effectively limiting transepidermal water loss and prolonging hydration. However, its poor skin penetration, high viscosity, and cohesive nature can hinder uniform and thin application, reducing its usability in topical formulations [8,24]. Increasing either concentration or MW of HA further elevates its viscosity, which complicates formulation development, reduces spreadability and skin absorption, and often results in a heavy, sticky texture that may diminish user acceptance.

The high viscosity of HA solutions is primarily attributed to their linear polysaccharide chains, which form extended and entangled molecular structures [25]. To address this limitation, various modifications have been explored to reduce viscosity without compromising performance. In general, branched polymer architectures have shown significantly lower viscosities because of reduced chain entanglement and smaller hydrodynamic volumes [26]. Many studies have aimed to improve the rheological properties of HA while preserving its moisturizing efficacy, including approaches such as incorporating bioactive substances and simply blending of high- and low-MW HAs [24,27,28]. Such HA-based materials are used not only in cosmetics but also in diverse medical applications, including wound dressings, dermal fillers, anti-adhesive barriers, osteoarthritis treatments, ophthalmic devices, and vesicoureteral reflux management [29,30]. Notably, low-viscosity HA offers excellent spreadability, tissue conformity, and sustained hydration, making it promising for skin-contact devices [31,32].

In this study, we aimed to decouple viscosity from the water-binding properties, enabling high performance at reduced viscosity levels. Previous studies have indicated that modified HA derivatives maintain or even enhance skin hydration properties while significantly improving the sensory profile of cosmetic formulations [33,34]. This study hypothesized that branched HA structures can significantly decrease solution viscosity without compromising the moisturizing efficacy, offering improved usability. We systematically investigated the synthesis of a branched HA hybrid (bHH) and evaluated its biocompatibility, physicochemical characteristics, rheological behavior, and moisturizing performance.

## 2. Results

### 2.1. Synthesis of bHH

**Figure 1:**
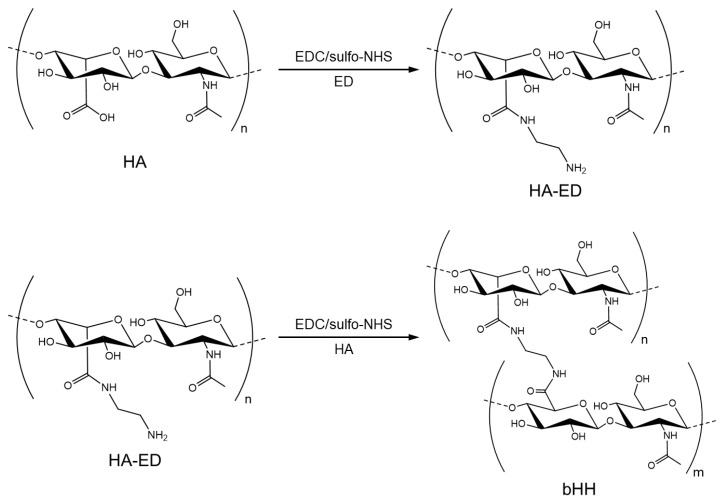
Synthetic scheme of bHH. HA was conjugated with ED as a linker (HA–ED) and then reacted with another HA to synthesize bHH.

The carboxyl group of HA was activated using 1-ethyl-3-(dimethylaminopropyl) carbodiimide (EDC) and *N*-hydroxysulfosuccinimide sodium salt (sulfo-NHS), followed by chemical conjugation with ethylenediamine (ED), which is a bifunctional linker, to obtain HA-ED. Another HA molecule was coupled with HA-ED in the presence of EDC and sulfo-NHS to synthesize bHH (Figure 1). Only 5 mol% of the carboxyl groups in the HA backbone was initially activated to preserve the native properties of HA and minimize the crosslinking density [35,36]. The reduction in the number of carboxyl groups in HA was determined by acid-base titration [37], and the conjugation efficiency of bHH synthesis was approximately 90%, regardless of the MW of HA in bHH. The numbers after HA and bHH indicate the MWs of the HA backbone.

**Figure 2:**
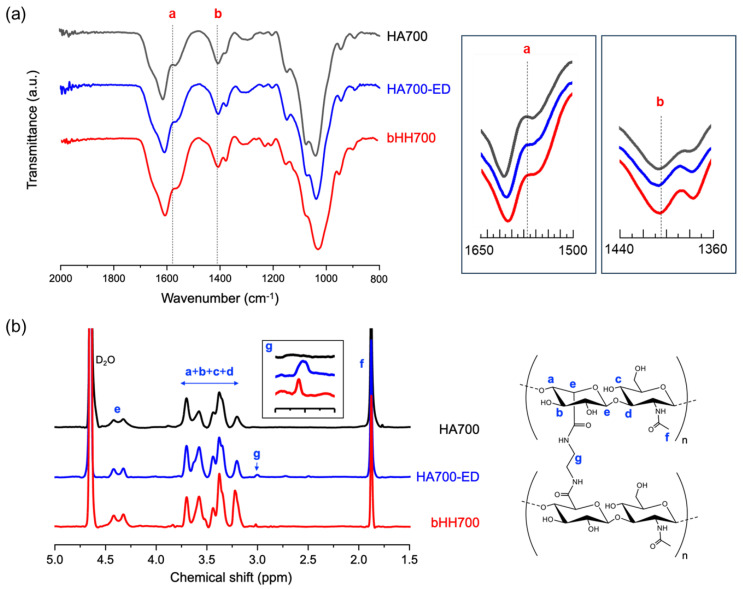
HA700, HA700-ED, and bHH700 characterized by (**a**) FT-IR and (**b**) ^1^H NMR spectroscopy. The raw spectra are presented without normalization and represent the actual values. In the FT-IR spectra, characteristic peaks at 1575 (**a**, N-H bending) and 1405 (**b**, COO^−^ stretching) cm^−1^ are labeled with red and shown enlarged in the insert boxes. In the ^1^H NMR spectra, proton signals are labeled with blue (**a**–**g**), with peak **g** at 3.0 ppm highlighted in an expanded insert box.

The synthesis of bHH was confirmed using Fourier transform infrared (FT-IR) and ^1^H nuclear magnetic resonance (NMR) spectroscopy. Representative spectra of bHH700 are presented (Figure 2). In the FT-IR spectra, both HA700-ED and bHH700 showed a reduction in peak intensity at 1405 cm^−1^ (COO- stretching, peak **b**), compared to HA700 (Figure 2a). This finding indicates consumption of carboxyl groups in glucuronic acid units during chemical modification. Additionally, an increase in peak intensity at 1575 cm^−1^ (N-H bending, peak **a**) was observed in the order HA700 < HA700-ED < bHH700, due to additional amide linkages formed during the coupling reaction. These assignments are consistent with previous reports, where COO^−^ stretching and N-H bending peaks have also been observed in modified HA and its hydrogels [38,39,40]. The formation of branched structure of bHH700 was further investigated using ^1^H NMR spectroscopy (Figure 2b). The ethylene group of HA700-ED exhibited a proton peak at 3.0 ppm, confirming successful conjugation of ED to HA700. The relative peak intensity of the ethylene group in bHH700 (normalized to the acetyl group peak at 1.9 ppm) decreased to 37% compared to HA700-ED, indicating substantial consumption of ED during the coupling reaction for the branched structure formation. These observations are consistent with previous findings describing characteristic proton signals in HA derivatives functionalized with amine linkers [41].

### 2.2. Rheological Properties of bHH

**Figure 3:**
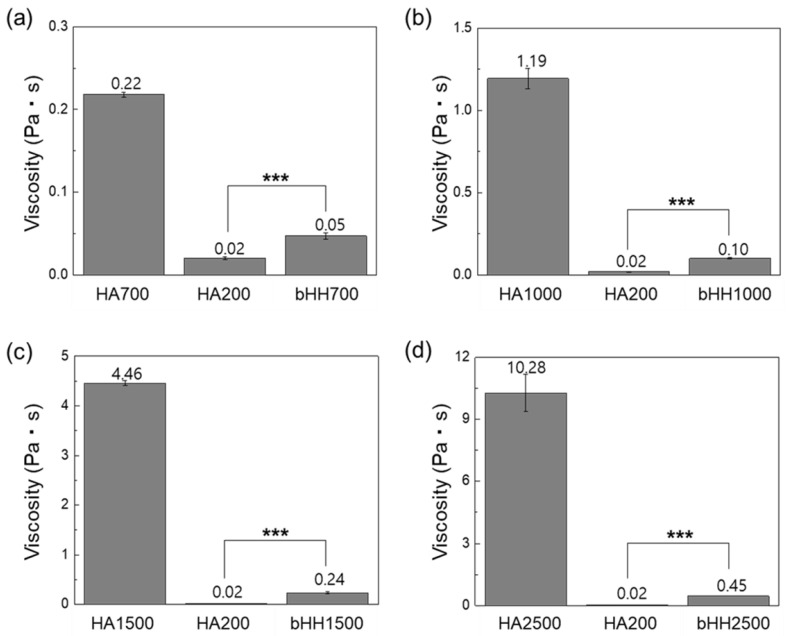
Solution viscosity of linear (**a**) HA700, (**b**) HA1000, (**c**) HA1500, and (**d**) HA2500. HA200 was coupled to the backbone of linear HA to synthesize bHH ([polymer] = 1 wt%, distilled water, 37 °C; mean ± standard deviation, n = 4, Student’s *t*-test, *** *p* < 0.001).

Comparisons of solution viscosity between linear HAs of various MWs (HA700, HA1000, HA1500, and HA2500), low-MW HA (HA200), and branched HA hybrids (bHH700, bHH1000, bHH1500, and bHH2500) are shown (Figure 3). The viscosity measurements were conducted at a polymer concentration of 1 wt% in distilled water at 37 °C. These results demonstrate that branching significantly reduced the viscosity of high-MW linear HA. Specifically, HA700, the linear form, exhibited a viscosity of 0.218 ± 0.003 Pa·s, whereas bHH700 exhibited a significantly lower viscosity of 0.047 ± 0.004 Pa·s (*p* < 0.001). HA1000 also exhibited a dramatic decrease in viscosity upon branching, with bHH1000 exhibiting a significantly lower viscosity than linear HA1000. The difference became even more pronounced with higher MWs for HA1500 and HA2500, where the linear forms had very high viscosities, which decreased dramatically in their branched hybrid forms (bHH1500 and bHH2500).

**Figure 4:**
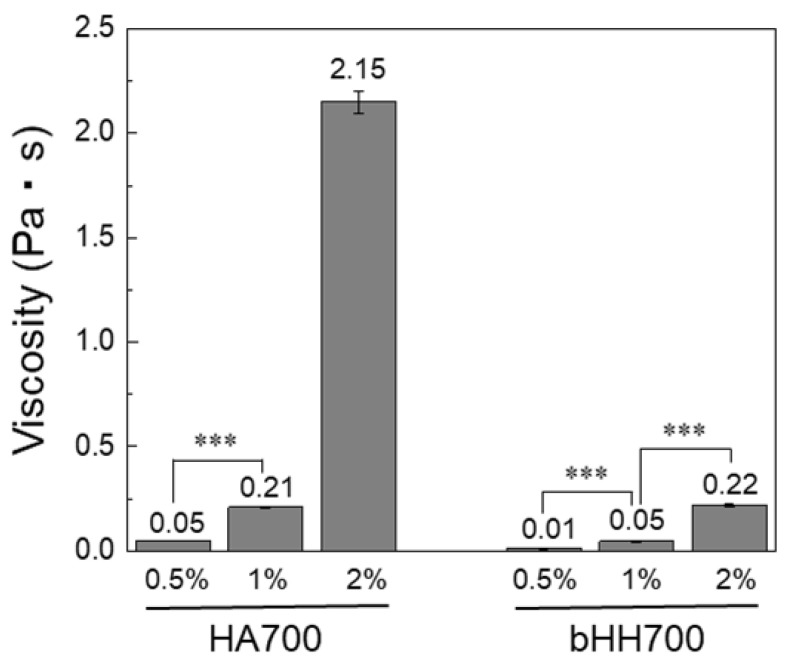
Concentration-dependent viscosity of HA700 and bHH700 (distilled water, 37 °C; mean ± standard deviation, n = 4, Student’s *t*-test, *** *p* < 0.001).

**Figure 5:**
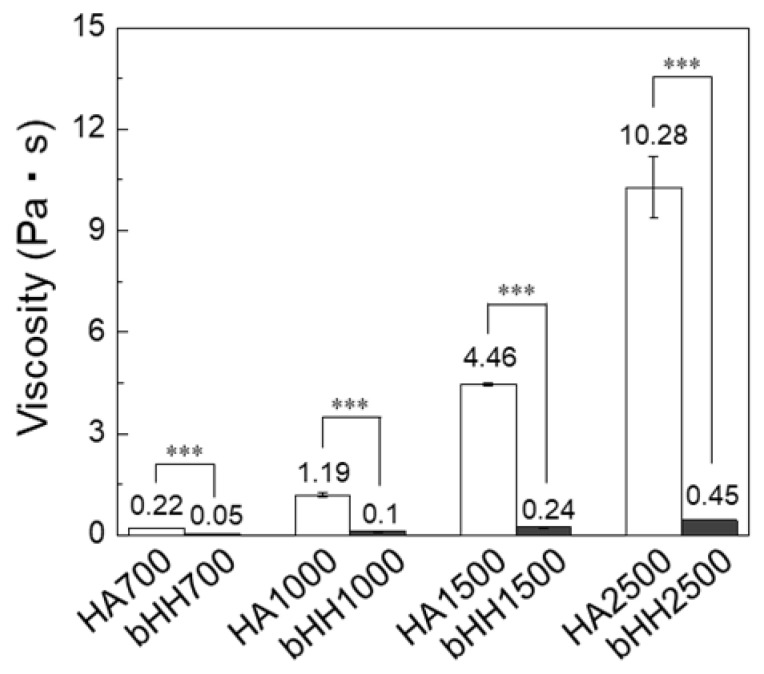
Viscosity of linear HA vs. bHH with the same molecular backbone and concentration ([polymer] = 1 wt%, distilled water, 37 °C; mean ± standard deviation, n = 4, Student’s *t*-test, *** *p* < 0.001).

A concentration-dependent viscosity comparison between linear HA and branched HA hybrid was next investigated. bHH700 was selected as a representative sample because higher-MW linear HAs exhibited limited solubility and excessive viscosity at concentrations above 1%, making uniform gel preparation and reliable viscosity measurements difficult. Linear HA700 showed a significant increase in viscosity with increasing polymer concentration, reaching approximately 2.15 Pa·s at 2%. In contrast, branched bHH700 consistently maintained a much lower viscosity across all tested concentrations, with only a slight incremental increase in viscosity, even at the highest tested concentration of 2%, where the viscosity was 0.22 Pa·s, compared with 0.5% or 1% (*p* < 0.001) (Figure 4). This indicated that branching effectively mitigated the concentration-dependent increase in viscosity typical of linear HA. The viscosities of all bHH synthesized with HA of different MWs were consistently lower than those of their linear HA counterparts. This viscosity reduction was more significant for higher-MW branched structures (Figure 5).

### 2.3. Moisturizing Effect (Ex Vivo)

**Figure 6:**
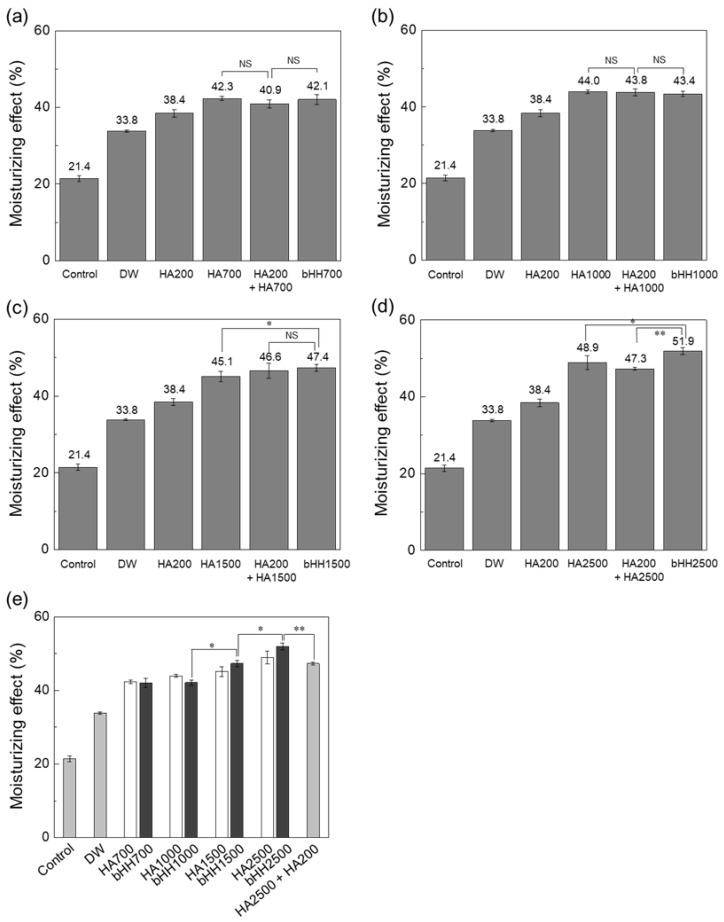
Moisturizing effect of HA and bHH with various MWs. HA200 was coupled to the backbone of linear (**a**) HA700, (**b**) HA1000, (**c**) HA1500, and (**d**) HA2500 to synthesize bHH. The moisturizing effect of simple mixtures was also tested. (**e**) Comparison of moisturizing effects of linear HA and branched bHH with various MWs. A simple mixture of HA2500 and HA200 was also included (DW, distilled water only; [polymer] = 0.5 wt%, 37 °C, 1 h; mean ± standard deviation, n = 4, Student’s *t*-test; NS, not significant; * *p* < 0.05, ** *p* < 0.01).

**Figure 7:**
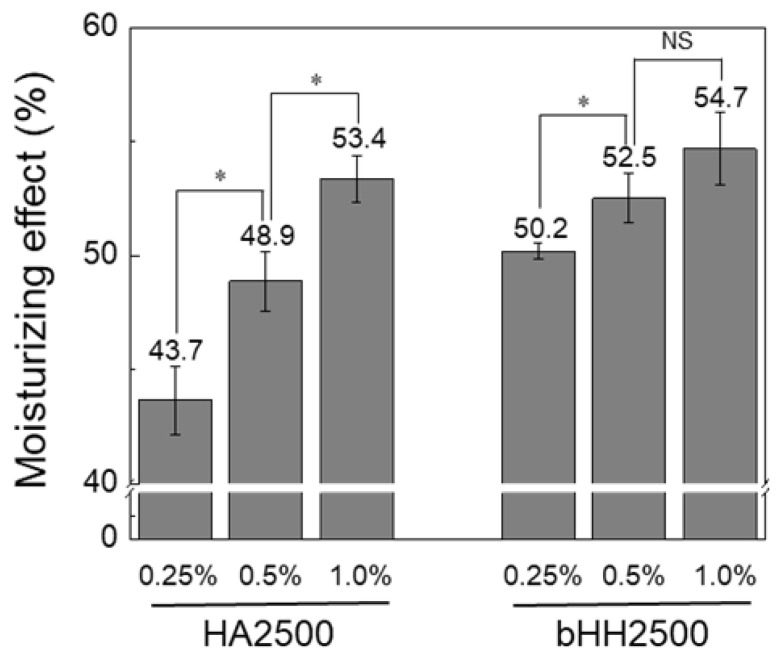
Moisturizing effect of HA2500 and bHH2500 at different concentrations (37 °C, 1 h; mean ± standard deviation, n = 4; Student’s *t*-test; NS, not significant; * *p* < 0.05).

The moisturizing effects of various HA formulations, including linear HA with different MWs (HA700, HA1000, HA1500, and HA2500), low-MW HA (HA200), simple physical mixtures, and branched HA hybrids (bHH700, bHH1000, bHH1500, and bHH2500), are shown in Figure 6. The efficacy was measured and compared between the control (untreated) and distilled water groups. Both linear HA (HA700, HA1000, HA1500, and HA2500) and their branched counterparts (bHH) exhibited significantly greater moisturizing effect than the control and distilled water groups. Notably, the branched forms maintained or slightly improved the moisturizing effect compared with their linear counterparts and simple mixtures with HA200 (Figure 6a–d). A comparative overview clearly indicated that higher MW formulations generally provide an increased moisturizing effect. Specifically, bHH2500 demonstrated a higher moisturizing effect (51.9 ± 0.9%) than linear HA2500 alone and the physical mixture of HA2500 and HA200 (47.3 ± 0.4%), indicating a synergistic effect owing to branching (*p* = 0.001) (Figure 6e). Overall, these results indicate that introducing branching structures into HA not only effectively reduces the solution viscosity but also maintains or enhances the moisturizing capacity, offering superior performance in skincare applications. Moreover, the moisturizing effect of bHH increased with increasing concentrations (Figure 7). HA2500 and bHH2500 are selected and shown in Figure 7, as the improvement in water-binding capacity was most evident at this molecular weight. This trend is consistent with the dose-dependent hydrating behavior of HA [42].

**Figure 8:**
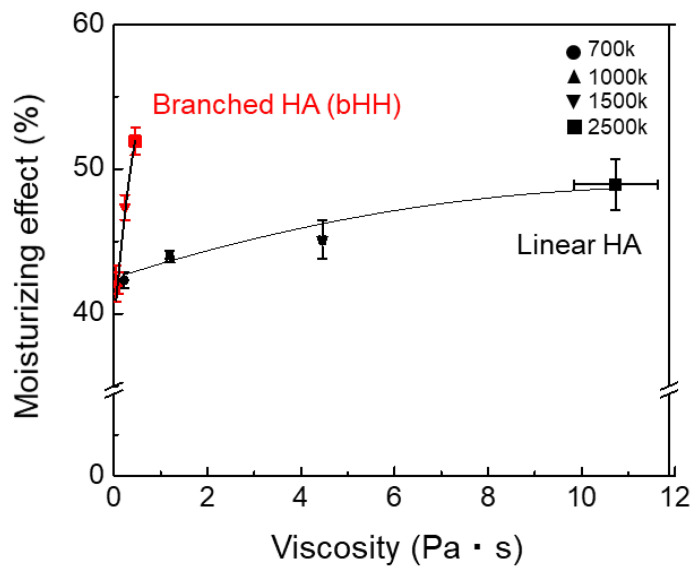
Moisturizing effect vs. solution viscosity for linear and branched HAs (mean ± standard deviation, n = 4).

The relationship between the moisturizing effect and solution viscosity of the linear and branched HAs was next investigated. Branched HAs consistently exhibited high moisturizing effect while maintaining low viscosity. In contrast, linear HAs demonstrated increased moisturizing effect associated with a significantly higher viscosity (Figure 8). Although similar trends were observed across all molecular weights, the effect became increasingly evident with higher MW. These findings highlight that branched structures successfully decouple moisturizing effect from viscosity, providing enhanced skin hydration at considerably reduced viscosities compared with their linear counterparts. The pH values of HA and bHH solutions dissolved in distilled water were measured to be approximately 6, which is near-neutral and generally considered suitable for topical skin application [43].

### 2.4. Cytotoxicity of bHH

**Figure 9:**
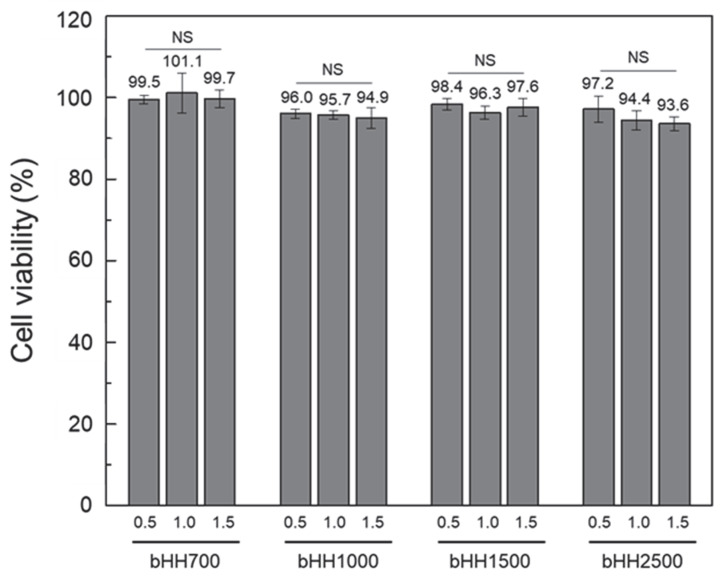
Relative viability (to cell-only control) of human dermal fibroblasts treated with various concentrations of bHH solutions ([polymer] = 0.5−1.5 mg/mL, 37 °C, 24 h; mean ± standard deviation, n = 4, Student’s *t*-test; NS, not significant).

The viability of human dermal fibroblasts treated with bHH solutions (bHH700, bHH1000, bHH1500, and bHH2500) at different concentrations was assessed in vitro relative to a cell-only control ([bHH] = 0.5–1.5 mg/mL). All bHH samples exhibited good preliminary biocompatibility, with no significant cytotoxicity observed (Figure 9). It should be noted, however, that these findings are limited to in vitro assays, and comprehensive in vivo studies will be required to establish definitive safety. Nevertheless, the results were consistent with previous reports that HA and its derivatives exhibit excellent safety profiles both in vitro and in vivo [36,37]. Collectively, these data support the potential suitability of bHH for topical and injectable applications in dermatological and cosmetic products.

## 3. Discussion

In this study, we demonstrated that the introduction of a branched structure into HA does not alter the inherent biocompatibility of linear HA. The MW of HA critically influences its water-binding capacity. Generally, high-MW HA retains more water than low-MW HA at equivalent concentrations. Notably, the hybrid HA exhibited significantly reduced solution viscosity, even at high concentrations, while maintaining its moisturizing performance. Introducing side chains or branches into polymers disrupts the linear structure and diminishes the entanglement of the polymer chains. Consequently, the flow resistance of branched polymers is lower than that of linear polymers at the same concentration, resulting in reduced viscosity. The ability to control viscosity without reducing polymer content is particularly advantageous for developing high-concentration formulations with favorable spreadability and textures.

The choice between small molecules and macromolecules may depend on specific product goals, desired consumer sensory experiences, and target skin conditions. Combining both categories may leverage the rapid effects of small molecules and sustained benefits of macromolecules, thereby enhancing the overall product performance and consumer satisfaction.

The low-viscosity HA formulations developed in this study demonstrate significant potential for a wide range of biomedical and cosmetic applications. Previous studies have reported that introducing branched or alternative architectures can decouple viscosity from hydration, allowing formulations to maintain moisturizing efficacy while reducing solution viscosity [44,45]. Among the branched HA hybrids, increasing their molecular weight allows for optimization of material properties: higher-MW bHH retains more water while maintaining lower viscosity compared with linear HA of a similar MW, effectively separating moisturizing efficacy from solution viscosity. This approach improves spreadability, minimizes stickiness, and enhances practical usability in topical and transdermal products. Selection of the molecular weight should be guided by the specific application, as all bHH variants possess potential utility within appropriate formulation contexts.

Due to its excellent skin conformity, spreadability, and biocompatibility, low-viscosity HA is particularly well-suited for use in skin-contact medical devices. These include products designed to protect sensitive tissues—such as inflamed skin or dry mucosa—while providing sustained hydration and supporting tissue repair and regeneration [46,47]. In addition, the favorable biocompatibility and injectability of HA enable its incorporation into a variety of advanced delivery platforms, including topical formulations, wound dressings, transdermal drug delivery systems, and wearable therapeutic devices. These systems benefit from HA’s ability to retain moisture over extended time periods and to facilitate the efficient delivery of bioactive compounds [47,48,49]. Importantly, the pH of aqueous HA and bHH solutions was measured near 6, which is suitable for skin application, and could be further adjusted using biocompatible buffers to optimize stability and minimize irritation [43].

By selecting appropriate MWs and concentrations, bHH formulations can be tailored to sustain moisturizing efficacy while reducing viscosity, supporting versatile and practical design for both cosmetic and biomedical applications.

## 4. Materials and Methods

### 4.1. Materials

Sodium hyaluronate with MWs of 200, 700, 1500, and 2500 kDa was purchased from Lifecore (Chaska, MN, USA), and sodium hyaluronate with an MW of 1000 kDa was obtained from Humedix (Anyang, Republic of Korea). 2-(*N*-morpholino)ethanesulfonic acid (MES), sodium chloride (NaCl), and ED were purchased from Sigma-Aldrich (St. Louis, MO, USA). EDC was purchased from Proteochem (Hurricane, UT, USA). Sulfo-NHS was purchased from CovaChem (Loves Park, IL, USA).

### 4.2. Synthesis of bHH

Each HA (MW *=* 700, 1000, 1500, 2500 kDa) was dissolved separately in a 0.1 M MES buffer solution (1 wt%, pH 6.0, 0.3 M NaCl) and reacted with ED in the presence of EDC and sulfo-NHS ([COOH]:[EDC]:[sulfo-NHS] = 1:0.1:0.05, mole ratio). After 20 h, the mixture was precipitated by adding absolute ethanol (five volumes relative to the reaction volume) and incubating at room temperature for 30 min. The resulting precipitate was collected, briefly air-dried, and lyophilized to obtain the HA-ED conjugate.

HA (MW = 200 kDa) was dissolved in 0.1 M MES buffer solution (1 wt%, pH 6.0, 0.3 M NaCl) containing EDC and sulfo-NHS ([COOH]:[EDC]:[sulfo-NHS] = 1:0.1:0.05, mole ratio) and added dropwise to the HA-ED solution (1 wt%, pH 6.0, 0.3 M NaCl) to synthesize bHH. After 20 h, the solution was precipitated with ethanol and lyophilized as described above. The bHH was synthesized at a weight ratio of 1:1.

### 4.3. Characterization of bHH

The synthesis of bHH was confirmed by FT-IR spectroscopy (Nicolet 6700, Thermo Fisher Scientific; Waltham, MA, USA). FT-IR spectra were recorded in the wavenumber range of 800–2000 cm^−1^ (resolution, 4 cm^−1^; scan rate, 4 mm/s). The synthesis of bHH was further verified by ^1^H NMR spectroscopy (D_2_O; VNMRS 600 MHz, Varian, Palo Alto, CA, USA). The residual water (HDO) peak was observed at 4.6–4.7 ppm.

### 4.4. Cell Viability Evaluation

Human dermal fibroblasts (ATCC, Manassas, VA, USA) were seeded onto a 96-well tissue culture plate at a density of 5 × 10^3^ cells/well and cultured in Dulbecco’s modified Eagle’s medium containing 10% fetal bovine serum and 100 units/mL penicillin-streptomycin (Gibco, Grand Island, NY, USA) at 37 °C under a 5% CO_2_ atmosphere. Cell viability was evaluated after 24 h of culture using an Ez-Cytox assay kit (Dogen, Daejeon, Korea) according to the manufacturer’s instruction. The absorbance was measured at 450 nm using a spectrophotometer (SpectraMax M2e, Molecular devices, Sunnyvale, CA, USA), and the cell viability was calculated relative to that of untreated cells.

### 4.5. Viscosity Measurement

The complex viscosity of bHH dissolved in distilled water was measured using a rotational rheometer (Bohlin Gemini 150; Malvern) with a cone-and-plate fixture (4° cone angle, 40 mm diameter plate, 800 µm gap from the bottom plate). All measurements were performed at 37 °C.

### 4.6. Water Retention Test (Ex Vivo)

Porcine skin samples (3 × 3 cm) were prepared by removing subcutaneous fat and equilibrating at 37 °C to assess the moisturizing effect. Each test sample (100 µL) was applied to the skin surfaces. After 1 h incubation at 37 °C, moisture content was measured using a skin moisture checker (MY-808S, Scalar, Japan). The moisturizing effect (%) was calculated based on the change in skin capacitance before and after treatment [24].

### 4.7. Statistical Analysis

All experiments were performed in quadruplicates (n = 4). Data are presented as mean ± standard deviation. Statistical significance was assessed using a Student’s *t*-test, with * *p* < 0.05, ** *p* < 0.01, and *** *p* < 0.001 considered statistically significant.

## 5. Conclusions

Branched structures were introduced into the HA backbone to manipulate the solution viscosity of high-MW HA while maintaining its moisturizing effectiveness. Coupling high- and low-MW HAs to form branched structures significantly reduced the solution viscosity, particularly at higher polymer concentrations; this is because of the decreased hydrodynamic volume of the polymer. High-MW linear HA exhibited enhanced water retention in an MW-dependent manner, whereas branched HAs maintained their moisturizing effect, and bHH2500 showed improved water-binding capacity compared with a simple mixture of HA2500 and HA200 with the same polymer content. Thus, the introduction of branched structures can successfully reduce the viscosity of high-MW HA without compromising its moisture properties. In vitro cytotoxicity assays indicated no significant toxicity of bHH formulations. These results demonstrate that branching effectively reduces viscosity while maintaining—or even enhancing—ex vivo moisturizing capacity. However, further in vivo studies are necessary to confirm both moisturizing performance and biocompatibility. This novel approach can prospectively enable the development of new formulations with improved utility and skin hydration, thereby contributing to advancements in both skincare and medical device technology.

## Data Availability

The raw data supporting the conclusions of this article will be made available by the authors on request.
